# Organic Foods Purchase Behavior among Generation Y of Bangladesh: The Moderation Effect of Trust and Price Consciousness

**DOI:** 10.3390/foods10102278

**Published:** 2021-09-26

**Authors:** Guang-Wen Zheng, Nazma Akter, Abu Bakkar Siddik, Mohammad Masukujjaman

**Affiliations:** 1 School of Economics and Management, Shaanxi University of Science and Technology (SUST), Weiyang University Park, Weiyang District, Xi’an 710021, China; zhengguangwen@sust.edu.cn; 2 School of Business, Ahsanullah University of Science and Technology, Tejgaon Industrial Area, Dhaka 1208, Bangladesh; nazma.sob@aust.edu; 3 Department of Business Administration, Northern University Bangladesh, Banani C/A, Dhaka 1213, Bangladesh; masukujjaman@nub.ac.bd

**Keywords:** Generation Y, intention–behavior gap, organic products, price consciousness, trust

## Abstract

This study aims to identify the factors influencing the purchase behavior of organic foods among young generation customers in Bangladesh. The study adopted the theory of planned behavior as a base and developed 11 hypotheses based on the extant empirical literature. Adopting the purposive sampling method, the primary data were obtained from a cross-sectional sample of 464 young Bangladeshi consumers using a survey method. In order to identify the key relationship among the study variables, the structural equation modeling (SEM) approach was employed using AMOS software, version 25. The study employed a purposive sampling method to pick young respondents through online media. The study revealed that health consciousness, environmental consciousness, food safety consciousness, price consciousness, novelty consciousness, and trust are factors that significantly affect purchase intention and subsequently, the actual purchase of organic foods. The novelty consciousness factor got the highest predicting power, followed by food safety concerns among Generation Y. The research also found that trust and price consciousness exhibit positive and negative moderating effects, respectively, on the relationship between purchase intention and actual purchase. However, the study did not find any moderating role of price consciousness on the association between environmental consciousness and purchase intention. As policy recommendations, informing and educating young consumers about organic products, their novelty, and other benefits of consuming is critical for fostering their purchase.

## 1. Introduction

Nowadays, people tend to be more aware of the social and ecological consequences of their purchases [1] and as a result, ethical consumerism is on the rise [2]. Ethical consumerism is the practice of purchasing items based on moral and personal beliefs as well as social factors, as opposed to solely economic considerations [3,4]. Buying organic foods is a form of ethical consumption, as it is produced without using chemical pesticides and herbicides, bioengineering, ionizing radiation, synthetic fertilizers, or sewage sludge in the production of these foods. Because of this, the global demand for organic foods has gradually but significantly increased [5], with sales estimated to have exceeded $90 billion in the last two decades [6].

Yet, the consumption of organic foods is not widespread among Bangladeshis. Despite possessing the ideal agricultural climate regions, organic food production is still a relatively recent development in Bangladesh. According to the Bangladesh Organic Products Manufacturers Association (BOPMA), Bangladeshi organic producers grow fruits, vegetables, dairy products, fruit juices, dried vegetables, and seafood [7]. A significant number of studies [8,9,10,11,12] exist on green consumer intention or behavior toward general products in Bangladesh. However, there is a dearth of research in the context of organic food products. Although [13,14] examined organic tea and organic food purchases, respectively, from the Bangladeshi perspective, it is necessary to conduct additional research on organic products to ascertain the reasons for their limited adoption and the various ways to accelerate their spread.

Understanding the green consumer behavior of Generation Y is critical in the design of green marketing strategies that are relevant to the target generation [15,16]. The Generation Y population are sometimes referred to as Millennials [17], Generation Me [18], or Echo Boomers [19] due to their “baby boomers offspring” status and the huge increase in birth rates between the early 1980s and mid-1990s (ages now 26–41 years). Generation Y individuals are highly sophisticated, technologically adept, and impervious to traditional marketing techniques. Additionally, they are more diverse in race and ethnic origins and tend to be more segmented in terms of media usage. Finally, owing to their rapid access to the internet, individuals have less brand loyalty and readily adopt new habits, styles, and means of communication [20]. According to researchers [21,22], Generation Y customers are typically enthusiastic about acquiring green products. Thus, the examination of this consumer segment will aid in the development of a more comprehensive realization of consumers of Generation Y and the associated issue of sustainable consumption. A study of Generation Y’s customer loyalty in banking [23] and their intention to green purchase decisions in green products is being conducted in Bangladesh [7]. However, to the researcher’s knowledge, no comprehensive paper exists on the consumer behavior of Generation Y on organic food items in Bangladesh.

Although the Theory of Planned Behavior (TPB) is popular, the intention–behavior gap is one of its shortcomings. Few empirical research studies [24,25,26] have explored the intention–behavior gap for organic products, and it can be concluded that some elements might impede the transition of intentions into behaviors [26]. Ismael and Ploeger [27] conducted their study from the perspective of a developed country (Germany), with subjective well-being as a moderator filling the gap. From the Bangladeshi perspective, trust regarding the authenticity of organic products is important in terms of the production process, its labeling, and its retailers, regarding whether they are supplying the original products or not. This is owing to the difficulty in finding genuine products among the several adulterated ones due to the lack of surveillance from regulatory authorities. Similarly, Nguyen et al. [28] assessed the attitude–behavior gap in Vietnam and suggested product availability as a moderator to fill the gap. During physical shopping, the availability of organic products was difficult and time-consuming to search and order from various shopping malls. In the COVID-19 pandemic, the e-commerce industry has become popular in Bangladesh and now online orders have become easier. It is just a click away from getting online grocery products, including organic foods. For this reason, organic product availability is not considered as a construct here in this research. Rather, lower prices became more important owing to the lower purchasing power of consumers amid the lingering pandemic. Thus, a gap exists in the literature, particularly from a developing country’s perspective.

To properly address such an apparent gap, the study researches the determinants of the green purchase decisions of organic food products based on generation Y consumers and assesses the moderation effects of trust and price consciousness between intention and actual behavior.

The remainder of the paper is split into seven parts: Section 2 outlines a review of the literature and the formation of hypotheses, Section 3 presents the methodology, Section 4 outlines the outcome of the research, Section 5 presents the results and discussion, Section 6 rounds off the theoretical part of the study, Section 7 fully highlights the policy implications, and the final section (Section 8) discusses the limitations and suggestions for future studies.

## 2. Literature Review

### 2.1. Theoretical Framework and Hypothesis Development

**Figure 1:**
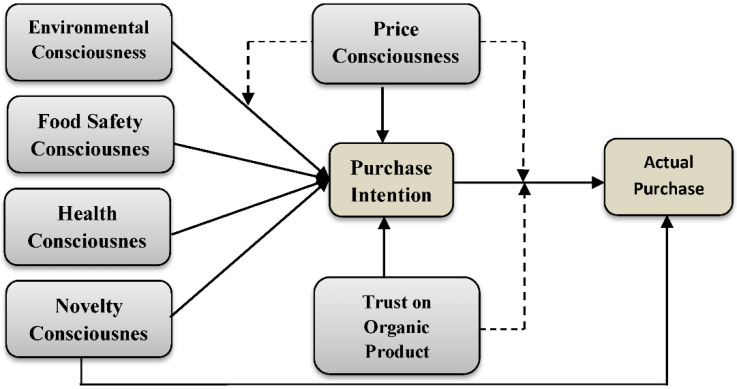
Conceptual Framework.

Specialized theoretical models such as the Theory of Reasoned Action (TRA) and the Theory of Planned Behavior (TPB) have been established to quantify and identify behavioral intention variables, particularly from an individual perspective. The TRA model of attitude–behavior research is well-known and widely used [29]. According to experts, the anticipation of future behavior based on prior acts is inadequate [30]. The TPB [31] is a well-known paradigm derived from the TRA that handles changeable perceived behavioral control. Ajzen and Fishbein [32] observed that values must be analyzed within the context of the situation at hand to anticipate specific behaviors. The intention–behavior gap is the limitation of the TPB theory, which has been addressed in this study, adding trust and price consciousness as moderator variables (Figure 1).

#### 2.1.1. Health Consciousness

The health consciousness (HC) of individuals depicts their attitude toward health issues, i.e., their willingness to take action to protect their health [33,34]. There is a widespread belief that organic foods are healthier because they are nutritionally dense and chemical-free [35]. According to Bryla [36], the essential characteristic of organic food for Polish customers is its healthfulness. Thus, health consciousness is a critical factor in the intake of organic foods [37]. Empirical data depicts that HC has a beneficial and influential effect on customers’ purchases and consumption intentions. Consumers who are worried about their health are more inclined to purchase organic foods than foods grown with non-organic methods [38,39,40]. The scholars [40,41,42] discovered that health consciousness is a more powerful motivator of the intention to purchase organic foods. As a result, the following hypothesis can be presented.

**Hypothesis** **1** **(H1).** *Health consciousness positively influences the purchase intention of organic foods*.

#### 2.1.2. Environmental Consciousness

Environmental consciousness refers to the extent to which individuals are aware of and willing to address environmental concerns [43]. Numerous environmental benefits have been ascribed to organic farming, including soil preservation, maintaining farm ecosystems, and ground and surface water protection [44,45]. According to Aman et al. [46] and Asif et al. [47], environmental concerns have a major impact on Sabahan consumers’ buying intentions (Table 1). While Xu et al. asserted that environmental consciousness has no direct effect on intention, Pagiaslis and Krontalis [48] discovered environmental concern to be directly and positively related to consumers’ intentions to purchase green products. Environmental concern is a crucial factor in assessing the desire to purchase organic food [49] since the purchase of organic foods is regarded as an environmentally friendly action. Thus, the more concerned a consumer is about the environment, the stronger their green purchasing intention and behavior [42]. Thus, the following hypothesis is proposed:

**Hypothesis** **2** **(H2).** *Environmental consciousness positively influences the purchase intention of organic foods*.

#### 2.1.3. Food Safety Consciousness

Food safety is a primary reason for the choice of organic foods [39]. The consumption of organic products mitigates the hazards connected with the consumption of chemically processed foods [56]. Owing to the health benefits of organic foods, safety concerns have been recognized as a significant motivator of purchase intention [57,58,59]. The concern of consumers towards food safety drives them to opt for organic products and tends to place higher premiums on local manufacturing [60]. The purchasers of organic products believe that their actions are of immense benefit to the surrounding residents [61] and their families [56]. Hence, we advance the following hypothesis:

**Hypothesis** **3** **(H3).** *Food safety consciousness positively influences the purchase intention of organic foods*.

#### 2.1.4. Price Consciousness

Price consciousness is the propensity of individuals to spend time and energy shopping for the best deals on (grocery) products [62]. According to prior research, people with strong levels of price sensitivity are less inclined to enforce their environmental knowledge and views on their intention to engage in green consumption. As observed by Hack [63], one of the primary reasons for people’s avoidance of organic foods is high costs. Willer et al. [6] discovered that organic foods are unpopular in underdeveloped nations due to their high costs. Katt and Meixner [42] discovered a negative association between price consciousness and organic foods purchase intention, resulting in our proposition of the following hypotheses:

**Hypothesis** **4** **(H4).** *Price consciousness negatively influences the purchase intention of organic foods*.

#### 2.1.5. Novelty Consciousness

The term “novelty consciousness” is regarded as an individual’s urge to purchase novel products [64]. A consumer’s desire to discover new products is a motivator that impacts their purchase patterns. Afshar Jahanshahi and Jia [9] discovered more conclusive evidence, suggesting that novelty consciousness (NC) has a significant impact on green purchasing patterns. They also observed a favorable correlation between the propensity of customers to opt for products that enhance their distinctiveness (creative choices) and green product-purchasing patterns in Peru and Bangladesh. Similarly, Lang et al. [65] discovered that customers in the United States with a high degree of creative freedom demonstrated high levels of openness in the acceptance of sustainable fashion goods or services (e.g., renting clothing, swapping, and clothing repairs). Thus the following hypotheses are postulated:

**Hypothesis** **5** **(H5).** *Novelty consciousness positively influences the purchase intention of organic foods*.

**Hypothesis** **6** **(H6).** *Novelty consciousness positively influences the actual purchase of organic foods*.

#### 2.1.6. Trust

Trust in organic foods is observed from the enforcement of high premiums on the taste, quality, certification, marketing, and production methods of organic foods, notwithstanding its inherent weaknesses. Trust in the production process of organic foods, including standards and control, has strong causal impacts on intention and behavior [66,67]. According to reports, a clear and visible label on the product is a prerequisite for organic food products [39]. Following the study conducted by Perrini et al. [68], trust in vendors among Italian consumers is significantly reliant on retailers’ commitments to customer’s rights and the environment. Trust has a significantly positive effect on a consumer’s buying intention [69] and has also been highlighted as a significant predictor of behavioral intention [70]. The purchasing and non-purchasing behaviors of consumers are significantly influenced by trust [71,72,73,74], which is particularly pronounced in the purchase of organic foods [67,72,75]. Yu’s [76] study indicated that customers’ trust has a substantial impact on their propensity to purchase organic products. As a result, the following hypothesis is developed:

**Hypothesis** **7** **(H7).** *Trust in labeling positively influences the purchase intention of organic foods*.

#### 2.1.7. Purchase Intention and Actual Behavior

Purchase intention, which represents the possibility of individual customers to purchase, is an important indicator of customers’ future consumption behaviors [77]. Due to the scarcity of behavioral data, only a few researchers have investigated the effects of intention on actual behavior [78]. Ajzen [79] asserted that intentions are the immediate results of actual behavior. Furthermore, Zeithaml [80] stated that the mediation of behavioral intentions has been extensively studied. According to the findings of Wee et al. [81], purchase intention has a considerable effect on actual behavior. The study [55] also highlighted a strong positive relationship between the intention to purchase organic foods and actual behavior, while Carfora et al. [82] observed the same trend for the purchase behavior of organic milk. Therefore, the following hypothesis is proposed:

**Hypothesis** **8** **(H8).** *Purchase intention positively influences the purchase behavior of organic foods*.

#### 2.1.8. The Mediating Role of Trust and Price Consciousness

Although studies indicate that the relationship between customer intention and actual behavior is consistent, this relationship can be further strengthened by incorporating certain moderators [83]. Harris and Hagger [84] argued that a customer may have the intention but still fail to act on it. The evidence suggests that a lack of trust is a significant barrier to the purchase of organic goods by consumers [67,85]. Sultan et al. [5] discovered that trust acts as a moderator in the intention–behavior gap.

Likewise, the primary reason for the non-purchase of eco-friendly food products by frequent purchasers is the cost [86]. In the case of Malaysia’s generation Y, price consciousness has been observed to be a mediator between the apparel interest and purchase intention of organic products [87]. The high cost of green foods is a variable contributing to the discord between the purchase intention of green foods and practices [86]. Additionally, as per prior research, people with high degrees of price sensitivity may be less inclined to impose their environmental knowledge and views on their intention to engage in green consumption. According to Yue et al. [53], price sensitivity did reduce the link between environmental concerns and green consumption intention, resulting in the undermining of the favorable effect of environmental concerns on green consumption intention.

**Hypothesis** **9** **(H9).** *Trust significantly moderates the intention–behavior relationship*.

**Hypothesis** **10** **(H10).** *Price consciousness plays a negative moderating role between organic purchase intention and behavior*.

**Hypothesis** **11** **(H11).** *Price consciousness negatively moderates between environmental consciousness and purchase intention*.

## 3. Research Methodology

This is an empirical study that utilizes a cross-sectional survey method. In this regard, the data were only obtained to assess the features of the population at a particular moment.

### 3.1. Sample and Data Collection Procedures

This study employed data from an online survey targeted at various online e-commerce sites such as Chaldal, Meena click, Khas food, and Direct Fresh. The study employed a purposive sampling method to pick young respondents. For this purpose, researchers created a messenger and WhatsApp group and sent them Google Docs question links individually after confirming their date of birth. As of 2021, the age group of 26–41 years is considered generation Y. The questionnaire was administered to 20 research experts, and before the collection of data, several changes were made depending on the pilot study recommendations. The current research gathered 464 completed responses (6.8% response rate), eliminating incomplete (206) and screened-out (140) responses. Although the response rate of the online surveys was lower than that of pen-and-paper and face-to-face, it was consistent with rates reported in the other studies [13].

The survey was taken voluntarily. We contacted them with the research’s background, aims, and methodology, as well as the benefits to managers and policymakers in the relevant disciplines. Therefore, we chose individuals who readily consented to join. They were informed that the information would be used strictly for academic reasons and would remain anonymous. The questionnaire also excluded information such as names, race, and religion, which could be used to discriminate. The study ensured respect for all respondents regardless of socio-economic status. Also, the research article did not appear to have used personal images, audio, video, or general information that would have violated the respondents’ rights. We only presented the processed data and made no particular opinions or statements.

### 3.2. Development of the Questionnaire

The data were obtained by administering a structured questionnaire. The questionnaire was adapted from several previous research studies and included 25 items. Health consciousness was assessed using the Nagaraj [52] and Asif [47] items, and three items were adapted from Sumi & Kabir [13] to measure trust in organic products. Novelty consciousness was measured with the three items adapted from the study by Afshar Jahanshahi & Jia [9]. Four items of food safety consciousness and environmental consciousness were also obtained from [54] and Nagaraj [52], respectively. Price consciousness was measured using the items from Prakash [21] and Katt & Meixner [42]. The purchase intention and actual purchases were adapted from Asif [47]. Some changes were made to the phrasing to suit organic food viewpoints. All items were measured on a 5-point Likert scale, with 1 indicating “strongly disagree”, and 5, “strongly agree.” In addition, demographic questions concerning the occupation, age, education, and gender of respondents were requested.

### 3.3. Statistical Analysis

The theoretical framework was investigated using SPSS and AMOS version 21. As suggested by Anderson and Gerbing [88], a two-stage Structural Equation Modeling (SEM) approach was employed. The first stage involved the conduction of the Confirmatory Factor Analysis (CFA) to determine the reliability and validity of the measurement model. The whole structural model was computed in the second stage to assess the overall model fit and postulated associations using the standardized regression coefficients (β) and *p*-values.

## 4. Results

### 4.1. Descriptive Statistics

The respondents’ demographic profiles are as follows: 324 males (69.8%) and 140 (30.2%) females. The majority of the respondents (190; 40.9%) were aged 25–34, followed by 29.1% in the age range 35–44, which indicates that the respondents were young. Most responders, i.e., 195, (42.0%) were graduates, followed by 107 (23.0%) and 87 HSC graduates (18.8 percent). The majority of respondents, or 245 (52.8%), were students, followed by 130 service holders (28%) and 89 entrepreneurs (19.2%).

### 4.2. Measurement Model

#### 4.2.1. Data Screening and Normality

Before the estimation model was implemented, the data were tested for outliers and normality to satisfy the normality assumption of the general linear model. To classify outliers, Cook’s distance was measured. Considering Steven’s [89] recommendation that responses with a Cook’s value greater than one are omitted, a total of eight outliers were excluded in the final analysis. In terms of normality, the results fit as the variance from normality was not an issue. The skewness and kurtosis values were less than ±3 and ±10 [90], respectively (Table 2).

#### 4.2.2. Reliability and Validity

The reliability values (Appendix A) exceed 0.70 for the confirmatory analysis of each factor utilizing the composite reliability, often referred to as Alpha in Cronbach’s alpha [91,92]. The Average Variance Extracted (AVE) describes the extent to which objects with constructs are mutually represented. Each AVE is considered ideal for construction if it has a minimum value of 0.50 [93]. Finally, the Fornell–Larcker test confirms that all factors exhibit sufficient discriminant validity. Statistics from the CFA indicate that survey instruments have the property of perfection in evaluating the intent to buy.

The discriminant validity of the constructs was calculated using the Fornell–Larcker criteria and Heterotrait–Monotraits ratio (HTMT). To assess a construct’s discriminant validity, the square root of its AVE value must exceed its highest correlation with any other construct in the model [94]. This is aligned with the outcomes of this study (Table 2).

Similarly, the HTMT, associated with the disattenuated construct score, tests the constructs’ connection (Table 3). Centered on a threshold value less than 0.9, this analysis concludes the absence of discriminant validity issues [95]. The study suggests that, on average, reliability and validity are satisfactory.

#### 4.2.3. Multicollinearity and Coefficient of Determination

The Variance Inflation Factor (VIF) is a useful technique to assess the presence of multicollinearity among independent variables, as suggested by Kleinbaum et al. [96]. The result (Table 3) indicates that the VIF varied between 1.240 and 1.853, exhibiting within a standard of one to five [97]. This indicates that multicollinearity was not a barrier in the advancement of this study to the next analysis.

Santosa [98] suggested the quantification of the model’s explanatory ability by evaluating the coefficient of determination of the endogenous variable (R^2^). Any value greater than 0.26 is considered higher, while any value less than 0.13 is considered weaker. Also, any value between these two levels is deemed to be mild [99]. As all the endogenous values discovered in this research pass the analysis’ prerequisites (see Table 2), and as suggested by Falk and Miller [100], the model is said to have a high degree of explanatory capacity.

### 4.3. Confirmatory Factor Analysis and Common Method Bias Testing

Based on the guidelines provided by Harman [101], common method bias was tested using Harman’s single-factor analysis approach that utilizes the exploratory factor analysis method. The single factor represented 27.5% of the variance in the factors, which is less than the 50% threshold. This affirms the absence of the common method bias.

In the measurement model, the confirmation of factors was examined using the Confirmatory Factor Analysis (CFA). The resulting CFA model produced good fit indices: Normed Chi-square (χ^2^/df) = 2.401 is less than 3 [102] and the Goodness of Fit Index (GFI) = 0.910, Adjusted Goodness-of-Fit Index (AGFI) = 0.911, Tucker–Lewis Index (TLI) = 0.924, Incremental Fit Indices (IFI) = 0.932, Comparative Fit Index (CFI) = 0.922, and Normed Fit Index (NFI) = 0.911 are above 0.90 [103]. The Root Mean Square Error of Approximation (RMSEA) value is 0.065, which is lower than 0.08 [104]. The t-values corresponding to all the items was significant at a rate of less than 5%.

### 4.4. Structural Modeling

Further to the conduction and passing of the CFA test, the structural model assessment was used to test the goodness of fit indices of the proposed model. The outcome of the SEM presented a well fitted data (χ^2^/df = 2.642, GFI = 0.906, CFI = 0.901, IFI = 0.912, NFI = 0.902; AGFI = 0.903). An RMSEA value of 0.069 was obtained, and this justifies the obtained cut-off value of less than 0.08 [104]. Other fit indices like the CFI, GFI, IFI, and NFI met the standard value of approximately 0.9 and higher [105]. As a result, the data in this study properly matches the model, as observed from the good fit of the indicators.

**Figure 2:**
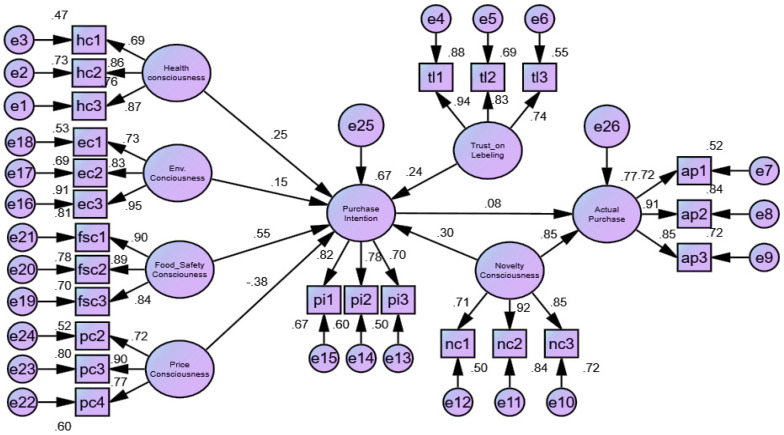
Structural model.

The study findings (see Table 4 and Figure 2) indicate the existence of statistically significant positive relationships between health consciousness (β = 0.249, *p* < 0.01), environmental consciousness (β = 0.150 *p* < 0.01), food safety consciousness (*β* = 0.547, *p* < 0.01), novelty consciousness (β = 0.298, *p* < 0.01), trust (β = 0.242, *p* < 0.01), and purchase intention. The perceived cost (β = −0.375, *p* < 0.01) also has a significant negative relationship with the purchase intention. Other significant relationships were observed to exist between purchase intention (β = 0.075, *p* < 0.05), novelty consciousness (β = 0.854, *p* < 0.01), and actual purchase. Thus, the outcomes corroborate Hypotheses 1–8.

### 4.5. Moderation Analysis

The moderation effect was assessed based on the interaction effects of the variables. The study results (Table 4 and Appendix B) show that trust (β = 0.127, t = 2.469, *p* < 0.05) and price consciousness (β = 0.103, t = 2.122, *p* < 0.05) have positive and negative moderating effects, respectively, on the association between purchase intention and actual purchase. In contrast, price consciousness does not moderate the association between environmental consciousness (β = −0.065, t = −1.534, *p* > 0.05) and the intention to buy. Therefore, hypotheses 9 and 11 are accepted, while Hypothesis 10 is rejected.

**Figure A1:**
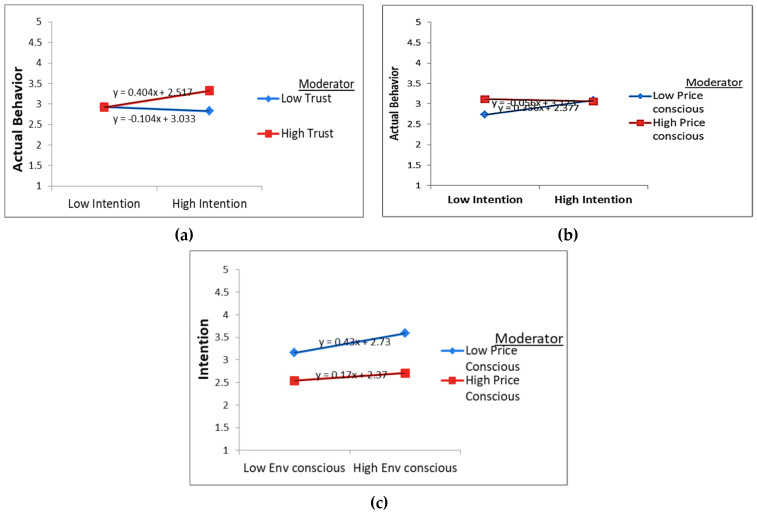
(**a**,**b**) Trust and price consciousness moderates intention-behavior (**c**) price consciousness do not moderate EC and PI relationships.

As illustrated in Figure A1, the impact of the interaction between price consciousness was plotted on a graph, and the slope of the low price-conscious group was observed to be steeper than that of the high price-conscious group. This depicts that the low price-conscious group’s purchase intention is more strongly linked with an actual purchase than it is for the high price-conscious group.

## 5. Discussion

As empirically established in this study, health consciousness affects the purchase intention of organic foods, thus validating the first hypothesis. This implies that Generation Y consumers prefer to purchase organic foods due to their health benefits. This result confirms the observation from past studies [42,47,52,89]. The study also corroborates Hypothesis 2, which postulates environmental consciousness as having a relationship with the purchase intention of organic foods, as those that are more environmentally conscious have a greater intention to purchase organic foods. Since Generation Y consumers are more interested in green products [21,22], the outcome is also in support of the Bangladesh context. However, this result is also is in line with past empirical studies [13,42,89] on organic products.

As per the result of this study, the third hypothesis (H3) of the existence of a significant relationship between food safety consciousness and the purchase intention of organic foods was supported, which is in accordance with the studies of Hsu et al. [58], Pino et al. [59], and Prentice et al. [57] but contrary to the study conducted by Nagaraj [52]. This signifies that people intend to purchase organic foods due to safety concerns as they perceive organic foods to be safer than other foods. Consistent with the study conducted by Katt & Meixner [42], the present study unveiled price consciousness as being negatively related to purchase intention. This implies that a higher price concern results in a lower purchase intention of organic products. The trust in organic products influences its purchase intention. This proposition is confirmed in this study. The trust in retailers also enhances the purchase intention of organic products. This result is aligned with the findings of Yu [86] and Sumi and Kabir [13], who observed trust to be a dominant factor predicting purchase intention.

As expected and in line with past works of literature [54,82], purchase intention is the driver of purchase behavior. This connotes that the greater the purchase intention, the greater the purchase behavior. Likewise, novelty consciousness was observed to be significantly related to actual purchases. Possessing novelty-seeking behavior tends to result in the purchase of organic foods since they are still fairly new to consumers. Young people are also sensitive to innovativeness, which is also proved for Bangladeshi Generation Y people. This result (H8) supports Afshar Jahanshahi and Jia’s [9] study, which holds a similar conclusion.

With respect to moderation effects, this study endorsed the moderating role of trust between intention and actual purchase. This result is in line with the study conducted by Sultan et al. [5], who discovered the moderating role of trust in the intention–behavior relationship. This implies that the stronger the trust a consumer holds to purchase organic foods, the higher their chances to purchase the product. In reverse, price consciousness plays a negative moderating role in the relationship between the purchase intention and actual purchase. In other words, as buying intentions increase, those having low price consciousness will be more likely to acquire organic products than individuals with high price consciousness. However, contrary to the study conducted by Yue et al. [53], the positive association between environmental concern and the intention to purchase did not have a moderating effect on price consciousness.

## 6. Conclusions

The study aimed to determine the factors affecting the purchase behavior of organic foods among the generation Y population in Bangladesh. The study revealed health consciousness, environmental consciousness, food safety consciousness, price consciousness, novelty consciousness, and trust as the factors that significantly affect the purchase intention of organic foods and subsequently, the actual purchase of organic foods. The research also observed that trust and price consciousness have positive and negative moderating effects, respectively, on the relationship between purchase intention and actual purchase. The study did not find any moderating role of price consciousness between environmental consciousness and purchase intention.

## 7. Implications

### 7.1. Theoretical Implications

This research has contributed to the extant literature in numerous ways. First, the present study worked on actual behavior instead of purchase intention as an endpoint. Thus, it caters to the limitation of many organic food research studies and contributes to the insights of actual purchase behavior from consumers’ perspectives. Second, the intention–behavior gap is the pressing issue experienced in individual behavior models, particularly for organic food products [104,106]. Consumers express a highly positive intention towards organic foods when probed, indicating a deficiency in intention–behavior, as many of these intentions do not transform into ultimate behavior. Sultan et al. [5] was the only scholar to have explored these gaps and outlined possible reasons for organic products’ purchase. The current study provides possible reasons for such gaps as well.

Third, the current study contributes to academia by establishing the moderating roles of price and trust between intention and actual behavior. It also establishes the moderation role of price consciousness in the intention–behavior gap. Existing research mainly regards price as a direct or indirect precedent of green procurement, while overlooking the moderating impacts of pricing consciousness in the consumption of organic products. To the best of our knowledge, this study is one of the first to empirically examine how price consciousness moderates the intention–behavior relationships. In addition, moderation of trust for an organic product is found to be significant from the Bangladeshi perspective. Although the moderating role of trust in the promotion of organic consumption behavior has attracted the attention of scholars [5] in developed countries, studies in developing countries are still scarce. This study stresses the importance of trust for developing countries, with an empirical investigation being particularly conducted to address the intention–behavior gap.

Fourth, the present study concentrates on the younger generation as respondents. Owens and Nowell [107] have commented that young consumers show an inclination towards emotional appeal rather than rationality, indicating the need for this age group to be separately studied to identify the emotional appeals, particularly to consumer behavior in Bangladesh. The study intends to paint a picture of the current young consumers’ views to inspire more precise and prominent directions for the future growth of organic products among consumers and businesses while also contributing to the body of knowledge on Generation Y as consumers and citizens.

### 7.2. Practical Implications

The outcome of this investigation has substantial practical relevance. To begin, previous research has indicated that customers who prefer organic food items place a lower emphasis on price [108]. However, this study’s findings indicated otherwise. In other words, we observed that Bangladeshi consumers place a higher concentration on price. Bangladeshi consumers generally compare the prices of regular food products to those of more expensive organic foods. Marketers could thus examine this issue and try to improve the value of organic foods by stressing the health benefits it offers to customers, thereby encouraging them to pay a small premium.

The results strengthen the claim that environmental and health concerns influence organic food purchasing intentions. Marketers should inform consumers about the health and ecological benefits of organic food, as these features remain the main incentive for the purchase of organic foods. The health and environmental concerns of consumers will make them resort to organic foods even if they cost more than normal meals [40]. Additionally, communication regarding health benefits is critical, as customers can connect them to their self-benefits.

Generation Y customers are more adaptable and willing to support efforts that benefit society [16]. Thus, informing and engaging Generation Y consumers about green behavior is critical for fostering a favorable attitude towards eco-friendly products and sustainability. Additionally, consumers that seek novelty during their shopping visits are more likely to buy green products. It is recommended that retailers and marketers emphasize the novelty of their green products and the manner it differs from regular items, as well as any revolutionary green technology that the product incorporates to increase sales.

As discovered in this study, trust has a moderating effect on the existing relationship between intention and behavior; educating customers on various organic certifications, production processes, packaging, and trustworthy retailers can be accomplished through mass communication and in-store communication. Certifying bodies could instill confidence in customers regarding the reliability of organic labeling. Such trust may spur consumers into purchasing organic foods that are properly labeled and certified.

## 8. Limitations and Research Direction

This study has certain shortcomings. First, the contextual factors of the organic product were only considered in the current research. Future studies could include cognitive factors such as self-efficacy, attitude, and perceived behavioral control, along with other factors considered in the model. Second, the current study concentrated on consumers who were generally younger and resided in metropolitan areas. The findings may differ from other age groups (Generation Z) or those residing in semi-urban and rural areas. Summarily, future research may focus on the following areas: first, cross-cultural studies to determine whether and how cultural gaps affect the consumption of organic foods. Second, comparable research should be conducted among customers residing in smaller towns and semi-urban areas, as consumers in such cities and towns are more likely to be exposed to locally produced foods and may have a more favorable attitude toward organic food purchases. Third, the current study only relied on the moderating variable in exploring the gap between intention and actual behavior and did not include any mediating variables. Upcoming research could consider mediating variables such as product availability, values, and attitude, etc., in examining the intention–behavior gap.

## Figures and Tables

**Table 1:** Empirical Literature on Organic Foods.

Context	Guiding Theory/DV	Sample Size/Methods	Significant Variables	Reference
Bangladesh	TPB/organic food purchase	337/SEM (AMOS)	Normative structure, trustworthiness, attitude, perceived behavioral control, and self-efficacy	[14]
Bangladesh	None/buying intention	174/PLS-SEM	Perceived price, perceived value, environmental concern, product attributes, health benefits, perceived quality, and buying intention	[13]
India	Theory of consumption values (TCV)/ethical consumption	452/SEM AMOS	Social value, emotional value, epistemic value, and ethical consumption intentions	[50]
India	None/purchase behavior of green products	Young people/SEM AMOS	Healthiness, price, brand, availability, certification, and eco-friendliness	[51]
India	None/purchase intention	438/SEM AMOS	Health consciousness, food safety concern, and consumer attitude	[52]
Pakistan, Turkey and Iran	None/intention to purchase	736/SEM AMOS	Attitude, health consciousness, subjective norms, perceived behavior control, and environmental concern	[47]
China	None/organic food purchase intention	680/SEM (AMOS)	Environmental concern, environmental responsibility, and price sensitivity	[53]
New Zealand	Reasoned action approach/purchase behavior	1052/SEM AMOS	PBC, actionable labeling, subjective norms, and intention	[54]
Norway	Big five personality model/organic food consumption	3501/Binary logistics regression	Extraversion, agreeableness, conscientiousness, emotional stability, and openness to experience	[55]

Note: TPB = Theory of Planned Behavior, SEM = Structural Equation Modeling, AMOS = Analysis of a Moment Structures, PLS = Partial Least Square.

**Table 2:** Correlation of Latent Variables and Square Roots of AVE.

	HC	EC	FSC	PC	TL	NC	PI	AP
Health consciousness (HC)	**0.809**							
Environmental consciousness (EC)	0.299 **	**0.842**						
Food safety consciousness (FSC)	0.391 **	0.339 **	**0.875**					
Price consciousness (PC)	−0.296 **	−0.212 **	−0.536 **	**0.801**				
Trust of labeling (TL)	0.192 **	0.165 **	0.326 **	−0.273 **	**0.841**			
Novelty consciousness (NC)	0.479 **	0.448 **	0.598 **	−0.414 **	0.298 **	**0.829**		
Purchase intention (PI)	0.503 **	0.431 **	0.738 **	−0.615 **	0.422 **	0.678 **	**0.769**	
Actual purchase (AP)	0.437 **	0.392 **	0.535 **	−0.370 **	0.206 **	0.792 **	0.606 **	**0.833**
Mean	3.638	3.798	3.464	2.122	3.353	3.208	3.277	3.313
Std. deviation	0.744	0.701	0.834	0.639	0.801	0.913	0.814	0.837
Skewness	−0.628	−0.400	−0.270	0.416	−0.265	−0.257	−0.220	−0.340
Kurtosis	1.150	1.090	−0.064	0.363	−0.213	−0.110	−0.354	0.168
R^2^	-	-	-	-	-	-	0.670	0.770

Note: Bold indicates the square root of AVE. ** Correlation is significant at the 0.01 level (2-tailed).

**Table 3:** Heterotrait–Monotrait Ratio (HTMT).

	HC	EC	FSC	PC	TL	NC	PI	AP	VIF	
									PI	AP
Health consciousness									1.240	-
Environmental consciousness	0.342								1.174	-
Food safety consciousness	0.442	0.380							1.604	-
Price consciousness	0.347	0.247	0.614						1.420	-
Trust of labeling	0.221	0.188	0.366	0.316					-	-
Novelty consciousness	0.553	0.514	0.675	0.486	0.339				-	1.853
Purchase intention	0.573	0.492	0.824	0.709	0.477	0.770			-	1.853
Actual purchase	0.501	0.446	0.600	0.434	0.231	0.891	0.682		-	-

VIF = Variance Inflation Factor.

**Table 4:** Structural Model and Hypothesis Testing Result.

Hypotheses	STD Beta	STD Error	*t*-Values	*p*-Values	Significance (*p* < 0.05)
H1: HC → PI	0.249	0.027	6.27 ***	0.000	Supported
H2: EC → PI	0.150	0.022	4.04 ***	0.000	Supported
H3: FSC → PI	0.547	0.030	11.69 ***	0.000	Supported
H4: PC → PI	−0.375	0.038	−8.63 ***	0.000	Supported
H5: NC → PI	0.298	0.023	7.49 ***	0.000	Supported
H6: TL → PI	0.242	0.024	6.27 ***	0.000	Supported
H7: PI → AP	0.075	0.046	2.271	0.023	Supported
H8: NC → AP	0.854	0.042	15.936 ***	0.000	Supported
H9: TL*PI → AP	0.127	0.058	2.469 **	0.017	Supported
H10: PC*EC → PI	0.065	0.053	1.534	0.094	Not supported
H11: PC*PI → AP	−0.103	0.072	−2.122 **	0.035	Supported

** Significant at 5% level, *** Significant at 1% level, STD = Standard, HC = health consciousness, EC = environmental consciousness, FSC = food safety consciousness, PC = price consciousness, TL = trust of labeling, NC = novelty consciousness, PI = purchase intention, AP = actual purchase.

## Data Availability

The data that support the findings of this study are available from the corresponding authors (A.B.S.) upon reasonable request.

## Appendix A

**Table A1:** Cronbach Alpha, Composite Reliability, AVE.

Constructs	Item Loading	Alpha α	CR	AVE
Health Consciousness [47,53]		0.833	0.849	0.655
I choose food carefully to ensure good health.	0.687			
As I go about my day, I am conscious of the state of my health.	0.857			
I often think about health-related issues.	0.871			
Trust [13]		0.869	0.877	0.707
Organic food labels are easily understood.	0.938			
Organic food certification is extremely reliable.	0.833			
Organic food promotion is credible.	0.739			
Novelty Consciousness [9]		0.862	0.867	0.687
It is great to purchase something fresh and exciting.	0.708			
By purchasing distinctive things or companies, I actively aim to create my personal uniqueness.	0.918			
I always buy new products when it is launched	0.847			
Environmental Consciousness [54]		0.872	0.878	0.709
I am a firm believer in nature and wildlife preservation.	0.726			
When making a majority of my purchases, I consider the potential environmental consequences.	0.830			
Human beings are severely abusing the environment.	0.954			
Food Safety consciousness [53]		0.907	0.908	0.766
I am concerned about the quality and safety of food today.	0.902			
Most foods now contain pesticide spray and fertilizer residues.	0.885			
The number of artificial additives and preservatives in food is highly worrying.	0.838			
I now purchase a greater variety of fresh fruits and vegetables than I did a few years ago.				
Price Consciousness [21,42]		0.839	0.842	0.641
The price of organic foods is relatively high.	0.722			
I attempt to purchase food goods that have discounts.	0.896			
I compare food prices from different brands.	0.774			
Purchase Intention [47]		0.885	0.812	0.591
If organic foods are available, I am interested in buying them.	0.821			
If organic foods are available, I intend to purchase them.	0.776			
I plan to purchase organic products during my next grocery shop.	0.704			
Actual Purchase [13]		0.877	0.871	0.694
I consume organic items regularly as healthy food.	0.722			
I have purchased “organic fruits” five times in the last five years.	0.915			
I choose to buy organic products even if I need to search for the next shop.	0.850			
